# Poster Session I – Poster of Distinction - A95 RESULTS OF A PROACTIVE REMOTE MONITORING QUALITY IMPROVEMENT (QI) INITIATIVE FOR PREGNANT PEOPLE WITH IBD

**DOI:** 10.1093/jcag/gwaf042.095

**Published:** 2026-02-13

**Authors:** M Harris, V Srikanth, K O’ Connor, M Al-Asafen, V W Huang

**Affiliations:** Sinai Health, Toronto, ON, Canada; Sinai Health, Toronto, ON, Canada; Sinai Health, Toronto, ON, Canada; Sinai Health, Toronto, ON, Canada; University of Toronto, Toronto, ON, Canada

## Abstract

**Background:**

Inflammatory Bowel Disease (IBD) during pregnancy requires specialized care. As part of a quality improvement (QI) initiative administered at the Mount Sinai Hospital Preconception and Pregnancy in IBD Clinic, we implemented proactive remote disease monitoring to improve maternal outcomes.

**Aims:**

1) To evaluate whether interventions performed in trimester 2 reduced mental health and disease activity scores in trimester 3.

2) To compare the trimester 3 scores between participants who received trimester 2 interventions and those who did not.

**Methods:**

Individuals with IBD under 27 weeks’ gestation were enrolled from August 2023 to September 2024 and were asked complete validated health status measurement tools per trimester and postpartum through an online dashboard. Responses that flagged increased disease activity (pMayo or mHBI), anxiety (GAD-7) or depressive symptoms (PHQ-9) triggered an intervention by an IBD nurse. Non-normal variables were summarized as medians (25^th^–75^th^%) and compared using Wilcoxon signed-rank tests for within-group analyses and Mann–Whitney U tests for between-group comparisons. A significance level of p < 0.05 was used.

**Results:**

Among participants who received an intervention in trimester 2, median GAD-7 and PHQ-9 scores decreased by trimester 3 but were significant for PHQ-9 only (*p*<0.01). Between group comparisons at trimester 3 showed anxiety and depression scores remained significantly higher among those who received trimester 2 interventions (*p*<0.001 for both). Within group changes in pMayo increased for those who did not receive an intervention in trimester 2 (*p*=0.001) whereas a significant decrease was observed for those who did (*p*=0.039). No significant between group differences were observed for disease activity indicators (mHBI and pMayo) at trimester 3.

**Conclusions:**

Proactive remote disease monitoring may help improve outcomes among pregnant IBD although further specialized mental health support amongst this population should be explored. Replication in larger in samples is recommended.

**Funding Agencies:**

Science of Care Institute - Sinai Health

